# How Water Interacts with the NOH Group: The Rotational Spectrum of the 1:1 N,N-diethylhydroxylamine·Water Complex

**DOI:** 10.3390/molecules27238190

**Published:** 2022-11-24

**Authors:** Giovanna Salvitti, Filippo Baroncelli, Chiara Nicotri, Luca Evangelisti, Sonia Melandri, Assimo Maris

**Affiliations:** 1 Department of Chemistry “G. Ciamician”, University of Bologna, 40126 Bologna, Italy; 2 Department of Chemistry “G. Ciamician”, Campus of Ravenna, University of Bologna, 48123 Ravenna, Italy; 3 Interdepartmental Centre for Industrial Aerospace Research (CIRI Aerospace), University of Bologna, 47121 Forlì, Italy; 4 Interdepartmental Centre for Industrial Agrifood Research (CIRI Agrifood), University of Bologna, 47521 Cesena, Italy

**Keywords:** water complex, microwave spectroscopy, hydrogen bond, quantum mechanical modelling, molecular structure, pulsed jet Fourier transform technique

## Abstract

The rotational spectrum of the 1:1 N,N-diethylhydroxylamine-water complex has been investigated using pulsed jet Fourier transform microwave spectroscopy in the 6.5–18.5 GHz frequency region. The most stable conformer has been detected as well as the ${}^{13}$C monosubstituted isotopologues in natural abundance and the ${}^{18}$O enriched water species, allowing to determine the nitrogen nuclear quadrupole coupling constants and the molecular structure in the vibrational ground state. The molecule has a $C_{s}$ symmetry and the water lies in the bc symmetry plane forming two hydrogen bonds with the NOH frame with length: $d_{\mathrm{HOH}\cdot \mathrm{NOH}}$ = 1.974 Å and $d_{H_{2}O\cdot \mathrm{HON}}$ = 2.096 Å. From symmetry-adapted perturbation theory calculations coupled to atoms in molecule approach, the corresponding interaction energy values are estimated to be 24 and 13 kJ·mol${}^{-1}$, respectively. The great strength of the intermolecular interaction involving the nitrogen atom is in agreement with the high reactivity of hydroxylamine compounds at the nitrogen site.

## 1. Introduction

Since its discovery [1], hydrogen bonding has been extensively investigated by both experimental and theoretical methods. Structural data arising from diffraction methods, magnetic resonance techniques, and rotationally resolved spectroscopies have been essential to understanding this non-covalent interaction which is ubiquitous in nature and plays an important role in chemistry and material science. Currently, according to IUPAC recommendation, the hydrogen bond is defined as an attractive interaction between a hydrogen atom from a molecule or a molecular fragment X–H in which X is more electronegative than H, and an atom or a group of atoms in the same or a different molecule, in which there is evidence of bond formation [2].

Perhaps, the most known example of hydrogen bonding formation is that of water, where hydroxyl groups act both as a proton donor and a proton acceptor creating an intermolecular network responsible for the peculiar physical and chemical properties of water, i.e., its high boiling point. However, water can bind also to hydroxyl groups belonging to different molecules, such as those of alcohols for instance. Also in these cases, water can exhibit different behaviors, that is water can act preferentially as a proton donor or proton acceptor with respect to the hydroxyl group of the partner molecule. Molecular beam rotational spectroscopy studies have shown that water usually acts as a proton donor when binding to alkyl alcohols (methanol [3], ethanol [4], *iso*-propanol [5], *tert*-butanol [6] *cis*-verbenol [7] and myrtenol [8]). Differently, when interacting with phenol [9] (${\mathrm{pK}}_{a}=9.88$) and *trans*-1,1,1,3,3,3-hexafluoro-2-propanol [10] (${\mathrm{pK}}_{a}=9.3$), which are weak acids, water acts as a proton acceptor.

Things become more complicated if the partner molecule is a bidentate ligand. If besides the hydroxyl group there is another substituent and they are close enough, water can bind to both of them, creating a closed structure. A planar 6-term cycle is formed when water binds to an acidic group, as in the case of sulfuric acid [11] nitric acid [12], formic acid [13], benzoic acid [14] and ibuprofen [15]. Interaction with ethanol derivatives leads to 6-term rings as in the case of 2-hydroxypyridine [16] or 7-term rings, as for 2-aminoethanol [17], glycidol [18], 2-fluoroethanol [19], 2,2,2-trifluoroethanol [20], *gauche*-1,1,1,3,3,3-hexafluoro-2-propanol [21], and propargyl alcohol [22]. In all of these molecular complexes, water assumes a role acting as a proton acceptor with respect to the hydroxyl group and as a proton donor with respect to the heteroatom (N, O, or F) or π bond. Differently, in the case of methyl salicylate, where the hydroxyl hydrogen atom is involved in an intramolecular interaction, water acts as a proton donor with respect to the hydroxyl group and as a proton acceptor with respect to a phenyl hydrogen atom [23].

**Figure 1:**
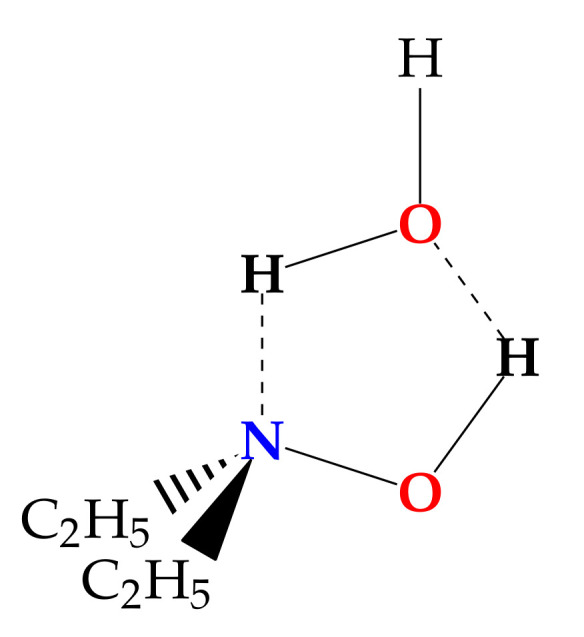
Sketch of DEHA-W.

Here we want to focus on a different kind of compound, where the hydroxyl group is directly bound to a heteroatom, making it possible to observe a 5-member ring structure. Indeed this arrangement has been observed in the water–hydroperoxy radical complex, whose structure is nearly planar [24]. It is worth noting that the calculated and experimental data provide different positions for the hydrogen atom of water not involved in the ring: out of, or in, plane, respectively. Other promising candidates able to bind water forming a 5-member ring are the hydroxylamine derivatives. To our knowledge, no rotational spectroscopy information is available about the interaction of water with the N-O-H functional group in the isolated phase. Therefore, we decided to study the monohydrate complex of N,N-diethylhydroxylamine (DEHA). DEHA is a hydroxylamine derivative where two ethyl chains replace the amino hydrogen atoms. Because of its volatility, low toxicity, and scavenger activity, it is used in the treatment of water [25,26]. Its properties in the isolated phase have been recently investigated by a mix of theoretical and spectroscopic techniques [27]. It has been found that twelve possible conformers exist depending on the orientation of the hydroxyl and ethyl groups. Using rotational spectroscopy, it has been proved that the most stable form corresponds to an overall *trans* arrangement of the alkyl chain and the hydroxyl eclipsing the nitrogen lone pair. This structure is well suited to provide both a proton donor (hydroxyl) and a proton acceptor (amine) site in the right orientation to bind a water molecule and create a 5-member ring structure (Figure 1). In the following, we report on the rotational spectrum of the 1:1 water complex of DEHA (DEHA-W) observed by pulsed jet Fourier transform microwave (PJ-FTMW) spectroscopy in the 6.5-18.5 GHz frequency range and the quantum mechanical investigation of its properties.

## 2. Materials and Methods

H${}_{2}^{18}$O (W18), purchased from Cambridge Isotopes Laboratories and DEHA (C${}_{4}$H${}_{11}$NO, CAS Registry Number: 3710-84-7) purchased from Merck with a declared minimum purity of 98%, have been used without further purification. DEHA is a colorless to light yellow liquid, miscible in water and with an ammoniacal odor. Declared properties are: vapor pressure 0.53 Pa at 273.15 K, melting point 247–248 K, boiling point 398–403 K, and refractive index 1.420 at 293 K.

The gas phase sample has been studied in supersonic expansion conditions, in which the molecules reach very low rotational temperatures, in the 6.5–18.5 GHz frequency region using a pulsed jet Fourier transform microwave (PJ-FTMW) spectrometer (COBRA-type [28,29]), whose details have been described previously [30]. Helium at a stagnation pressure of 0.3 MPa was passed over a container with water and subsequently, on a container DEHA, both kept at room temperature and expanded through a solenoid valve (General Valve, Series 9, nozzle diameter 0.5 mm) into the Fabry-Pérot cavity. The spectral line positions are determined after Fourier transformation of the time-domain signal with 8 k data points, recorded with 100 ns sample intervals. Each rotational transition appears as a doublet due to the Doppler effect. The line position is calculated as the arithmetic mean of the frequencies of the Doppler components. The estimated accuracy of the frequency measurements is better than 3 kHz and lines separated by more than 7 kHz are resolvable. The rotational temperature of the molecules in the pulsed jet was estimated to be about 1 K.

Minima on the conformational potential energy surface (PES) were determined by geometry optimization and subsequent evaluation of the Hessian matrix using the gaussian16^®^ software package (G16, Rev. C.01) (Gaussian is a registered trademark of Gaussian, Inc. 340 Quinnipiac St. Bldg. 40, Wallingford, CT 06492 USA). Preliminary calculations applied density functional theory (DFT) through the Becke-three-parameters Lee-Yang-Parr hybrid density functional theory (B3LYP) [31,32] corrected by the D3 version of Grimme’s empirical dispersion with Becke-Johnson damping (D3(BJ) [33,34] and combined with the valence triple-zeta quality Karlsruhe polarized type basis set (def2-TZVP) [35]. Subsequently, selected cases were further investigated at the *ab initio* level through the Møller-Plesset second-order perturbation theory (MP2) [36] in combination with the valence triple-zeta quality Dunning correlation consistent polarized type basis set augmented with diffuse functions (aug-cc-pVTZ) [37]. The theoretical electron density distributions were analyzed by means of Bader’s quantum theory of atoms-in-molecules [38] implemented in multiwfn program [39]. The intermolecular interaction energy has been evaluated through Symmetry-Adapted Perturbation Theory (SAPT) [40] using a high-order approach (DF-SAPT2+(3)δMP2/aug-cc-pVTZ//MP2/aug-cc-pVTZ) implemented in the psi4 package [41].

## 3. Results and Discussion

### 3.1. Conformational Analysis

**Figure 2:**
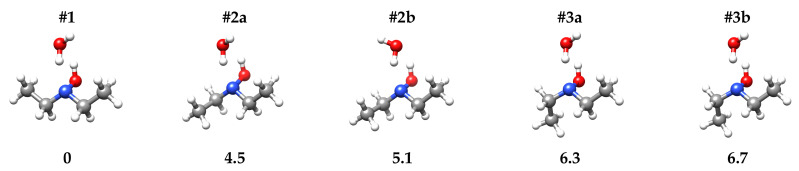
Sketch and zero-point corrected relative energy values ($\Delta E_{0}$ [kJ·mol${}^{-1}$]) of DEHA-W conformers.

Salvitti et al. [27] have found that the three most stable conformers of DEHA are characterized by the hydroxyl hydrogen atom being in *trans* orientation with respect to the bisector of the CNC angle and one of the methyl groups in *trans* orientation to the CNC frame. The other methyl group lies in the three staggered positions, namely *trans* (#1), *gauche* (#2) or *gauche*’ (#3). Conformer #1 is the global minimum, while the relative energy values of conformers #2 and #3 are estimated to be 4.6 and 6.5 kJ·mol${}^{-1}$, respectively, at the B3LYP-D3(BJ)/def2-TZVP level of calculation. Calculations performed at the same level of theory, suggest that the binding with a water molecule does not alter this order. The binding involves two hydrogen bonds where the DEHA hydroxyl group acts as a proton donor and the nitrogen lone pair acts as a proton acceptor forming a five-membered ring, with the hydrogen atom of water not involved in the hydrogen bond (H-free) located out-of-plane. Depending on the orientation of H-free, two different conformers are possible, the ones where H-free faces the methyl group (#2a and #3a) being more stable than the others (#2b and #3b) by about 0.5 kJ·mol${}^{-1}$, as shown in Figure 2. However, in the case of conformer #1, the two orientations lead to two equivalent species separated by a transition state where water lies on the bc-symmetry plane of the monomer. The interconversion barrier between the two equivalent conformers is calculated as 2.89 and 2.07 kJ·mol${}^{-1}$ at the B3LYP-D3(BJ)/def2-TZVP and MP2/aug-cc-pVTZ levels, respectively.

### 3.2. Rotational Spectrum

Preliminary rotational spectrum predictions were made using the calculated spectroscopic constants reported in Table 1. They allowed identifying several R-$\mu_{b}$- and R-$\mu_{c}$-type transition lines, reported in Table A1 and Table A2, respectively, characterized by a hyperfine structure arising from the quadrupole interaction of the ${}^{14}$N nuclear spin (*I* = 1) with the overall rotation, whereas no splitting arising from methyl internal rotation has been observed. The observed lines were assigned by direct diagonalization of a Watson *S*-reduced semirigid Hamiltonian in the ${\mathrm{III}}^{l}$-representation which includes an additional term to fit the nuclear hyperfine structure:

(1) $$\hat{H}={\hat{H}}_{R}+{\hat{H}}_{\mathrm{CD}}+{\hat{H}}_{Q}$$

where ${\hat{H}}_{R}$ represents the rigid rotor related to the *A*, *B*, and *C* rotational constants, ${\hat{H}}_{CD}$ considers the quartic centrifugal distortion effect, and ${\hat{H}}_{Q}$ is the operator associated to the nuclear quadrupole coupling interaction.

**Figure 3:**
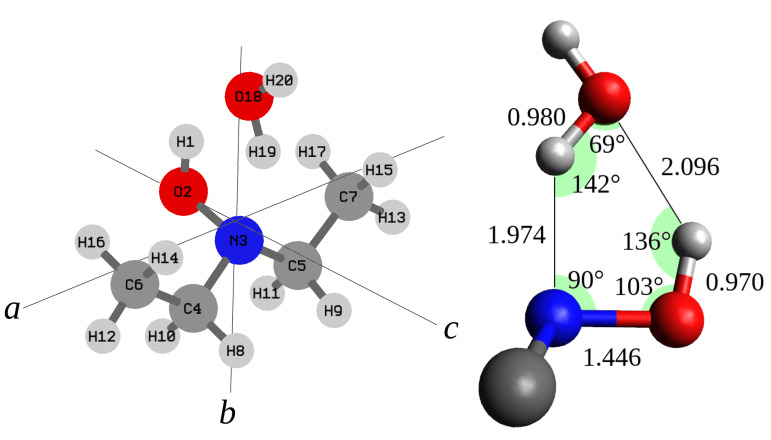
Left side: structure, number of the atoms, and principal inertial axis system of DEHA-W. Right side: $r_{0}$ bond distances (Å) and angles.

The fitting procedure was carried out using calpgm program suite [42] and the obtained constants are reported in Table 2. Comparison between the experimental and theoretical rotational constants and nuclear quadrupole coupling constants shows that the best match is with the most stable conformer (#1) (Figure 3). According to the calculated electric dipole moment components of DEHA-W#1, the $\mu_{c}$-type transition lines are more intense than the $\mu_{b}$-type ones. However, also the $\mu_{a}$-type transition lines were expected to be observed, but, despite careful searching, they could not be detected. This suggests that the $\mu_{a}$ electric dipole moment component is very small or even zero, which is compatible with $C_{s}$-structure, where the NOH-H${}_{2}$O frame lies in the bc-symmetry plane.

To confirm such an idea, we looked for the features of the monosubstituted ${}^{13}$C isotopologues. Indeed, depending on the $C_{1}$ or $C_{s}$ symmetry of the molecular structure, four or two sets of lines are expected to be observed, respectively. Only two sets of R-$\mu_{c}$-type transition lines were detected (Table A3), whose intensities with respect to the parent species lines are about double (≃2%) of the ${}^{13}$C natural abundance (≃1.1%), further confirming the $C_{s}$ hypothesis.

As regards the distribution of the masses in space, it must be pointed out that in the $C_{s}$ hypothesis the planar moment of inertia along the *a*-inertial axis ($M_{\mathrm{aa}}=\sum_{i=1}^{\mathrm{atmos}}m_{i}\cdot a_{i}^{2}$) should be equal for the monomer and the water complex. The value of the hydrated form exceeds the reference value by 1.05 uÅ${}^{2}$. Although this difference is not negligible, it could be ascribed to the effect of the intermolecular large amplitude motions on the vibrational ground state rotational constants, in agreement with an overall symmetric arrangement. To support this interpretation, additional measurements were conducted using ${}^{18}$O enriched water. This has allowed assigning several R-$\mu_{b}$- and R-$\mu_{c}$-type transition lines to DEHA-W18 (Table A4). From these results we can demonstrate that the $M_{\mathrm{aa}}$ of DEHA-W18 is only 0.006 uÅ${}^{2}$ larger than that of DEHA-W, probing that at least the oxygen of water lies in the bc-inertial plane. The derived spectroscopic constants of all the observed isotopologues are also listed in Table 2.

It is worth noting, that DEHA-W is a very asymmetric rotor, Ray’s asymmetry parameter $\kappa =\frac{2B-A-C}{A-C}$ [43] being almost zero. In particular, DEHA-W is a slightly prolate top (κ = −0.098), whereas DEHA-W18 is a slightly oblate top (κ = +0.052). For all the species, the more reliable fitting has been obtained using the ${\mathrm{III}}^{l}$-representation instead of the $I^{r}$-representation which provided negative values for the quartic centrifugal distortion constant $D_{J}$.

### 3.3. Molecular Structure

Direct information on the molecular structure can be obtained by applying Kraitchman’s substitution method [44]. Kraitchman’s equations [44] provide the so-called substitution structure ($r_{s}$), that is a set of coordinates, in absolute values, for all isotopically monosubstituted atoms relative to the principal inertial axes system of the parent species, under the assumption that the isotopic substitution does not alter the geometry. The obtained substitution coordinates with Costain’s errors [45] for the water oxygen atom and DEHA carbon atoms are compared to the theoretical equilibrium values ($r_{e}$) in Table 3. It may be noted that unlike the $a_{e}$ predicted coordinate, the $|a_{s}|$ substitution coordinate of the water oxygen atom is very close to zero. Moreover, the carbon atoms substitution coordinates do not match with either of the theoretical values, being halfway between them. These considerations, further support the hypothesis of an effective symmetric arrangement of the complex, as for the water-hydroperoxy radical complex [24].

Thus, assuming a $C_{s}$ symmetry, the structure of DEHA-W#1 has been optimized at the MP2/aug-cc-pVTZ level by constraining the whole water molecule to lie on the *bc*-plane. The achieved geometry is reported in Table 4 and has been used as starting point to perform a least-squares structural fit where a set of selected coordinates has been adjusted to reproduce the twelve rotational constants of the four observed species with a maximum discrepancy of 0.21 MHz. The strfit [46] software has been used and the results obtained by changing the CC and CN skeletal bond distances, the intermolecular hydrogen bond distance between the water oxygen and the hydroxyl hydrogen atom and the O${}_{W}\cdot$OH angle are summarized in Table 4 and visualized in Figure 3. The data show that the length of the two hydrogen bonds is about 2 Å, the N·H${}_{w}$ bond ($d_{N\cdot H_{w}}$ = 1.974 Å) being 0.12 Å shorter than the H·O${}_{w}$ bond ($d_{H\cdot O_{w}}$ = 2.096 Å). Interestingly the behavior is reversed in the case of the water-hydroperoxy radical complex (H${}_{2}$O·HO${}_{2}$) where the O${}_{w}\cdot$H bond is shorter ($d_{H\cdot O_{w}}$ = 1.875 Å) than the O·H${}_{w}$ bond [24]. As expected, we observe that the presence of two hydrogen bonds makes the two subunits of the complex closer with respect to each single hydrogen bond. Actually the O·O distance ($d_{O\cdot O}$ = 2.877 Å) is shorter by about 0.10 Å than the that of water dimer ($d_{O\cdot O}$ = 2.976 Å [47]) and the O·N distance ($d_{O\cdot N}$ = 2.811 Å) is about 0.05 Å shorter than that of quinuclidine-water complex [48].

### 3.4. Intermolecular Interaction Energy

Experimental results show that in DEHA-W the N·H${}_{W}$ hydrogen bond is shorter than the H·O${}_{W}$, suggesting that the former is stronger than the latter. To quantify this effect, QTAIM was applied to the theoretical MP2/aug-cc-pVTZ electron density values at the equilibrium geometry ($r_{e}$). With this approach, a chemical bond is characterized by a special point called the bond critical point (BCP) for which the electron density function between two nuclei is a minimum. The values of electron density at the inter-molecular BCPs are given in Table 5. The value for the hydrogen bond involving the nitrogen atom is about 1.8 times that of the hydrogen bond involving the oxygen atom. Assuming that the electron density at the BCP reflects the strength of the hydrogen bond, Emamian et al. [49] proposed the following formula to estimate the interaction energy in neutral compounds:

(2) $$E_{\mathrm{int}}/\mathrm{kcal}\cdot {\mathrm{mol}}^{-1}=-223.08\cdot \rho +0.7423$$

The resulting values, also given in Table 5, show that the N·H${}_{W}$ interaction energy is almost twice that of H·O${}_{W}$. The sum of these two values is about 43.9 kJ·mol${}^{-1}$ similar to the *ab initio* value 42.5 kJ·mol${}^{-1}$.

A more suitable approach for the estimation of the total interaction energy is provided by SAPT which considers the total interaction energy as a perturbation to the total system energy and is made free of basis set superposition error in a natural way [40]. Using a high-order SAPT approach (DF-SAPT2+(3)δMP2/aug-cc-pVTZ//MP2/aug-cc-pVTZ) we obtained the results listed in Table 5. The interaction energy value is the lowest one, −37.4 kJ·mol${}^{-1}$. It is worth noting that the 5.1 kJ·mol${}^{-1}$ displacement between the interaction energy values calculated with the QTAIM and SAPT approach is far from negligible, in contrast to what it has been determined in the case of 2-aminoacetophenone, where the water molecule binds to the acetyl group, for which the shift is less than 1 kJ·mol${}^{-1}$ [50]. However, the approach of Emamian et al. [49] for the estimation of the interaction energy from the electron density at the BCP has been designed for single hydrogen bond interactions. Therefore, considering the SAPT value as the best estimation and by applying the ratio obtained with QTAIM, we can attribute −24 and −13 kJ·mol${}^{-1}$ to the N·H${}_{W}$ and H·O${}_{W}$ hydrogen bonds, respectively.

## 4. Conclusions

The rotational spectrum of the parent species of DEHA-W and three isotopologues have been detected and assigned for the first time using FTMW spectroscopy, allowing to determine the molecular structure of the complex which exhibits a $C_{s}$ symmetry and two hydrogen bonds involving a hydroxyl hydrogen atom. It is worth noting that at the equilibrium geometry the hydrogen atom not involved in the hydrogen bond is predicted to lie out of plane, leading to two equivalent non-symmetric species. The same behavior has been found for another complex where water is involved in a 5-member intermolecular ring structure the water–hydroperoxy radical complex [24].

With the support of quantum mechanical calculations, it has been possible to determine that the hydrogen bond where the electronegative acceptor atom is nitrogen is shorter and stronger than the hydrogen bond where the electronegative acceptor atom is oxygen. This result is in agreement with the reactivity shown by hydroxylamine compounds. For example, the alkylation of hydroxylamines with diphenylmethane [51] shows that the nitrogen site is more nucleophilic than the oxygen one. Actually, *O*-alkylation only occurs if particular electron-withdrawing groups are used to deactivate the nitrogen lone pair and even in this case the *O*-alkylation does not become preferential and the final product is a mixture of *N*,*O*-alkylated and *N*,*N*-alkylated hydroxylamines, with a major abundance of the second product. 

## Figures and Tables

**Table 1:** Theoretical energy values and spectroscopic parameters of DEHA-W conformers.

			DFT ^a^			MP2 ^b^
	#1	#2a	#2b	#3a	#3b	#1
$E_{e}$ [Ha]	−365.589953	−365.588184	−365.587936	−365.587679	−365.587484	−364.762353
$E_{0}$ [Ha]	−365.412107	−365.410396	−365.410170	−365.409708	−365.409570	−364.582764
$D_{e}$ [kJ·mol${}^{-1}$]	−44.21	−44.20	−43.54	−44.54	−44.03	−40.83
$D_{0}$ [kJ·mol${}^{-1}$]	−33.41	−33.52	−32.92	−33.65	−33.29	−30.64
*A* [MHz]	2583.040	2248.239	2323.442	2631.291	2690.333	2603.301
*B* [MHz]	1819.991	1813.067	1767.119	1728.857	1706.002	1832.838
*C* [MHz]	1290.482	1361.421	1360.637	1255.979	1258.110	1297.150
κ	−0.18	0.02	−0.16	−0.31	−0.38	−0.18
$M_{\mathrm{aa}}$ [uÅ${}^{2}$]	236.825	212.584	219.953	251.317	255.041	235.606
$M_{\mathrm{bb}}$ [uÅ${}^{2}$]	154.795	158.630	151.475	151.062	146.656	154.001
$M_{\mathrm{cc}}$ [uÅ${}^{2}$]	40.857	66.159	66.038	41.003	41.194	40.129
$\mu_{a}$ [D]	1.369	−0.480	−2.295	−0.195	−1.531	1.316
$\mu_{b}$ [D]	−1.071	−1.669	0.262	−1.911	0.353	−0.907
$\mu_{c}$ [D]	1.612	1.698	1.099	1.389	1.905	1.585
$\mu_{\mathrm{tot}}$ [D]	2.371	2.429	2.558	2.370	2.470	2.251
$\chi_{\mathrm{aa}}$ [MHz]	0.759	1.239	1.103	−3.999	−3.877	0.513
$\chi_{\mathrm{bb}}$ [MHz]	−4.076	−1.920	−2.004	0.162	−0.008	−3.684
$\chi_{\mathrm{cc}}$ [MHz]	3.317	0.681	0.901	3.837	3.885	3.171

^a^ B3LYP-D3(BJ)/def2-TZVP. ^b^ MP2/aug-cc-pVTZ.

**Table 2:** Experimental spectroscopic parameters of DEHA-W in the *S*-reduction and ${\mathrm{III}}^{l}$-representation.

	Parent	${}^{13}$C6	${}^{13}$C4	${}^{18}$O18
*A* [MHz]	2500.1146(5) ^a^	2498.7482(5)	2483.7333(7)	2356.9534(5)
*B* [MHz]	1824.6348(5)	1785.2392(9)	1814.4391(11)	1823.9622(5)
*C* [MHz]	1269.6279(5)	1250.2201(35)	1261.0218(55)	1231.9071(6)
$D_{J}$ [kHz]	5.56(1)	5.83(3)	5.87(5)	5.84(1)
$D_{\mathrm{JK}}$ [kHz]	−10.14(4)	[0] ^b^	[0]	−10.87(5)
$D_{K}$ [kHz]	4.72(5)	[0]	[0]	5.24(5)
$d_{1}$ [kHz]	0.43(1)	[0]	[0]	0.40(2)
$d_{2}$ [kHz]	2.209(7)	[0]	[0]	2.376(9)
1.5$\chi_{\mathrm{cc}}$ [MHz]	4.873(3)	4.868(5)	4.915(7)	5.007(4)
($\chi_{\mathrm{bb}}$−$\chi_{\mathrm{aa}}$)/4 [MHz]	1.144(1)	−1.144(2)	−1.146(6)	−1.170(1)
σ [kHz] ^c^	3.3	5.3	3.8	3.5
*N* ^d^	79	18	11	56
μ-type ^e^	b, c	c	c	b, c
$M_{\mathrm{aa}}$ [uÅ${}^{2}$] ^f^	236.443	242.533	237.913	236.449
$M_{\mathrm{bb}}$ [uÅ${}^{2}$]	161.610	161.699	162.857	173.792
$M_{\mathrm{cc}}$ [uÅ${}^{2}$]	40.532	40.554	40.619	40.628
κ	−0.098	−0.143	−0.095	0.052
$\chi_{\mathrm{aa}}$ [MHz]	0.664(2)	0.665(3)	0.654(5)	0.671(3)
$\chi_{\mathrm{bb}}$ [MHz]	−3.912(3)	−3.911(6)	−3.930(15)	−4.009(4)
$\chi_{\mathrm{cc}}$ [MHz]	3.249(3)	3.245(6)	3.277(15)	3.338(4)

^a^ Error in units of the last digit. ^b^ Values in square brackets are fixed. ^c^ Standard deviation of the fit. ^d^ Number of lines in the fit. ^e^ Kind of observed rotational transitions. ^f^ Derived parameters.

**Table 3:** Substitution ($r_{s}$,$C_{s}$), ground state ($r_{0}$,$C_{s}$) and equilibrium ($r_{e}$,$C_{1}$) principal axis system coordinates of DEHA-W.

		$|r_{s}|$ [Å]	$r_{0}$ [Å] ^a^	$r_{e}^{\mathrm{DFT}}$ [Å] ^b^	$r_{e}^{\mathrm{MP}2}$ [Å] ^c^
O18	*a*	0.05(3)	0	−0.127	−0.435
	*b*	2.4806(6)	−2.4868(5)	−2.430	−2.396
	*c*	0.231(7)	0.268(2)	0.313	0.306
C4/C5	*a*	1.206(1)	±1.209(4)	1.243/−1.186	1.338/−1.062
	*b*	1.131(1)	−1.1355(1)	−1.100/−1.142	−1.001/−1.229
	*c*	0.298(5)	0.299(1)	0.284/0.302	0.286/0.293
C6/C7	*a*	2.4737(6)	±2.479(2)	2.486/−2.461	2.510/−2.395
	*b*	0.311(5)	−0.297(3)	−0.232/−0.324	−0.044/−0.517
	*c*	0.15(1)	0.1810(5)	0.198/0.192	0.198/0.172

^a^ The system has been constrained to Cs-symmetry. ^b^ B3LYP-D3(BJ)/def2-TZVP. ^c^ MP2/aug-cc-pVTZ.

**Table 4:** Theoretical structure ($r_{e}$) and derived $r_{0}$ parameters of DEHA-W in the symmetric ($C_{s}$) arrangement.

				$r_{e}$ MP2/aug-cc-pVTZ			$r_{0}$	
				$d_{e}$ [Å]	$\alpha_{e}{[}^{\circ }]$	$\tau_{e}{[}^{\circ }]$	$d_{0}$ [Å]	$\alpha_{0}{[}^{\circ }]$
O2	H1			0.97000				
N3	O2	H1		1.44541	102.932			
C${}_{5}^{4}$	N3	O2	H1	1.46504	105.535	∓121.393	1.470(4)	
C${}_{7}^{6}$	C${}_{5}^{4}$	N3	O2	1.51543	111.701	±69.156	1.526(6)	
H${}_{9}^{8}$	C${}_{5}^{4}$	N3	O2	1.09140	106.159	±120.524		
H${}_{11}^{10}$	C${}_{5}^{4}$	N3	O2	1.09697	109.299	∓122.999		
H${}_{13}^{12}$	C${}_{7}^{6}$	C${}_{5}^{4}$	N3	1.08948	110.017	±178.686		
H${}_{15}^{14}$	C${}_{7}^{6}$	C${}_{5}^{4}$	N3	1.08906	110.071	±58.705		
H${}_{17}^{16}$	C${}_{7}^{6}$	C${}_{5}^{4}$	N3	1.08730	110.534	∓61.602		
O18	H1	O2	N3	2.09161	133.847	0.000	2.097(3)	136.36(2)
H19	O18	H1	O2	0.98035	68.546	0.000		
H20	O18	H9	H1	0.96009	106.390	180.000		

**Table 5:** Intermolecular BCP’s electron density and interaction energy values of DEHA-W.

QTAIM ^a^	N·H${}_{W}$		H·O${}_{W}$		Global
ρ(r) [e/a${}_{0}^{3}$]	0.03445		0.01924		
$E_{\mathrm{int}}$ [kJ·mol${}^{-1}$]	−29.05		−14.85		−43.90
MP2/aug-cc-pVTZ					Global
$E_{\mathrm{int}}$ [kJ·mol${}^{-1}$]					−42.51
SAPT ^b^	Electrostatic	Exchange −repulsion	Induction	Dispersion	Global
$E_{\mathrm{int}}$ [kJ·mol${}^{-1}$]	−74.99	95.46	−27.28	−30.61	−37.42

^a^ Calculation performed on the MP2/aug-cc-pVTZ electron density. ^b^ DF-SAPT2+(3)dMP2/aug-cc-pVTZ//MP2/aug-cc-pVTZ.

## Data Availability

The data presented in this study are available within the article.

## Appendix A

**Table A1:** Experimental transition lines of DEHA·H${}_{2}$O.

$J^{\prime \prime }$	$K_{a}^{\prime \prime }$	$K_{c}^{\prime \prime }$	$F^{\prime \prime }$	$J^{\prime }$	$K_{a}^{\prime }$	$K_{c}^{\prime }$	$F^{\prime }$	ν [MHz]	o.−c.
2	2	0	1	1	1	1	0	9551.229	0.0070
2	2	0	3	1	1	1	2	9553.209	−0.0005
2	2	0	2	1	1	1	2	9553.617	−0.0008
2	2	0	1	1	1	1	1	9554.159	0.0018
2	2	0	2	1	1	1	1	9554.795	0.0032
2	2	1	2	1	1	0	1	8768.853	−0.0043
2	2	1	1	1	1	0	1	8769.188	−0.0016
2	2	1	2	1	1	0	2	8769.826	−0.0061
2	2	1	3	1	1	0	2	8770.047	0.0016
2	2	1	1	1	1	0	0	8771.618	−0.0086
3	0	3	2	2	1	2	1	8195.298	0.0058
3	0	3	4	2	1	2	3	8195.534	0.0005
3	0	3	3	2	1	2	2	8195.604	−0.0047
3	1	3	3	2	0	2	3	8675.577	0.0006
3	1	3	3	2	0	2	2	8675.983	−0.0017
3	1	3	4	2	0	2	3	8676.770	0.0017
3	1	3	2	2	0	2	1	8676.960	0.0009
3	2	2	3	2	1	1	2	11,307.726	0.0001
3	2	2	4	2	1	1	3	11,308.977	−0.0068
3	2	2	3	2	1	1	3	11,308.990	0.0062
3	2	2	2	2	1	1	1	11,309.683	0.0003
4	0	4	4	3	1	3	4	10,900.183	−0.0018
4	0	4	4	3	1	3	3	10,901.375	−0.0017
4	0	4	3	3	1	3	2	10,901.413	0.0018
4	0	4	5	3	1	3	4	10,901.492	−0.0002
4	1	4	4	3	0	3	3	11,074.083	−0.0011
4	1	4	5	3	0	3	4	11,074.484	0.0002
4	1	4	3	3	0	3	2	11,074.498	0.0013
4	2	3	4	3	1	2	3	13,571.615	0.0008
4	2	3	5	3	1	2	4	13,572.743	0.0025
4	2	3	3	3	1	2	2	13,573.131	0.0033
5	0	5	5	4	1	4	4	13,499.450	0.0001
5	0	5	4	4	1	4	3	13,499.542	−0.0004
5	0	5	6	4	1	4	5	13,499.587	−0.0006

**Table A2:** Experimental $\mu_{c}$-type transition lines of DEHA·H${}_{2}$O.

$J^{\prime \prime }$	$K_{a}^{\prime \prime }$	$K_{c}^{\prime \prime }$	$F^{\prime \prime }$	$J^{\prime }$	$K_{a}^{\prime }$	$K_{c}^{\prime }$	$F^{\prime }$	ν [MHz]	o.−c.
2	1	1	1	1	0	1	1	7972.751	0.0000
2	1	1	1	1	0	1	0	7973.247	−0.0016
2	1	1	3	1	0	1	2	7973.649	0.0004
2	1	1	2	1	0	1	1	7974.706	−0.0015
2	1	1	2	1	0	1	2	7974.906	−0.0005
2	2	0	1	1	1	0	1	8997.372	−0.0024
2	2	0	2	1	1	0	1	8998.007	−0.0020
2	2	0	3	1	1	0	2	8998.576	0.0006
2	2	0	2	1	1	0	2	8998.985	0.0013
2	2	0	1	1	1	0	0	8999.803	−0.0083
2	2	1	1	1	1	1	0	9323.045	0.0078
2	2	1	2	1	1	1	2	9324.465	−0.0012
2	2	1	3	1	1	1	2	9324.681	0.0015
2	2	1	2	1	1	1	1	9325.645	0.0049
2	2	1	1	1	1	1	1	9325.978	0.0056
3	1	2	2	2	0	2	1	11,940.401	−0.0023
3	1	2	4	2	0	2	3	11,940.997	−0.0003
3	1	2	3	2	0	2	3	11,942.045	−0.0021
3	1	2	3	2	0	2	2	11,942.456	0.0006
3	2	1	2	2	1	1	2	12,273.830	−0.0010
3	2	1	3	2	1	1	2	12,275.439	0.0009
3	2	1	4	2	1	1	3	12,275.507	0.0017
3	2	1	2	2	1	1	1	12,275.787	−0.0005
3	2	1	3	2	1	1	3	12,276.697	0.0011
3	2	2	2	2	1	2	1	12,972.865	−0.0022
3	2	2	3	2	1	2	3	12,973.444	−0.0037
3	2	2	4	2	1	2	3	12,973.454	0.0064
3	2	2	3	2	1	2	2	12,974.491	−0.0011
3	3	0	3	2	2	0	2	14,006.106	−0.0008
3	3	0	4	2	2	0	3	14,006.481	−0.0006
3	3	0	2	2	2	0	1	14,006.700	0.0035
4	1	3	3	3	0	3	2	16,219.589	−0.0071
4	1	3	5	3	0	3	4	16,220.081	−0.0004
4	1	3	4	3	0	3	3	16,221.627	0.0087
4	2	2	5	3	1	2	4	15,867.514	0.0019
4	2	2	4	3	1	2	3	15,867.893	0.0020
4	2	3	3	3	1	3	2	16,836.567	−0.0050
4	2	3	5	3	1	3	4	16,836.967	−0.0025
4	2	3	4	3	1	3	3	16,838.087	0.0021
4	3	1	4	3	2	1	3	17,024.305	0.0003
4	3	1	5	3	2	1	4	17,024.725	−0.0038
4	3	1	3	3	2	1	2	17,024.946	−0.0024
4	3	2	3	3	2	2	2	17,587.531	0.0033
4	3	2	5	3	2	2	4	17,587.593	−0.0020
4	3	2	4	3	2	2	3	17,587.857	0.0009

**Table A3:** Experimental $\mu_{b}$-type transition lines of ${}^{13}$C-DEHA·H${}_{2}$O.

$J^{\prime \prime }$	$K_{a}^{\prime \prime }$	$K_{c}^{\prime \prime }$	$F^{\prime \prime }$	$J^{\prime }$	$K_{a}^{\prime }$	$K_{c}^{\prime }$	$F^{\prime }$	ν [MHz] ${}^{13}$C${}_{6}$	o.−c.	ν [MHz] ${}^{13}$C${}_{4}$	o.−c.
2	1	1	1	1	0	1	0	7853.634	0.0001		
2	1	1	3	1	0	1	2	7854.033	−0.0001	7926.619	0.0045
2	1	1	2	1	0	1	1	7855.088	−0.0025	7927.677	−0.0048
2	2	0	1	1	1	0	1	8953.000	−0.0072	8939.850	−0.0015
2	2	0	2	1	1	0	1	8953.583	−0.0057	8940.502	0.0021
2	2	0	3	1	1	0	2	8954.186	−0.0019	8941.058	−0.0075
2	2	0	2	1	1	0	2	8954.565	0.0029		
2	2	0	1	1	1	0	0	8955.433	−0.0078	8942.313	0.0045
2	2	1	1	1	1	1	0	9279.518	0.0092		
2	2	1	2	1	1	1	2	9280.935	−0.0006		
2	2	1	3	1	1	1	2	9281.151	0.0019	9265.305	0.0038
2	2	1	2	1	1	1	1	9282.113	0.0046	9266.269	−0.0011
2	2	1	1	1	1	1	1	9282.448	0.0070		
3	2	1	3	2	1	1	2	12,137.770	0.0033	12,200.517	−0.0018
3	2	1	4	2	1	1	3	12,137.874	0.0032	12,200.586	0.0035
3	2	1	2	2	1	1	1	12,138.173	0.0074		
3	2	2	2	2	1	2	1	12,850.514	−0.0069		
3	2	2	4	2	1	2	3	12,851.097	−0.0035	12,893.647	−0.0017
3	2	2	3	2	1	2	2	12,852.140	−0.0035		

**Table A4:** Experimental transition lines of DEHA·H${}_{2}^{18}$O.

$J^{\prime \prime }$	$K_{a}^{\prime \prime }$	$K_{c}^{\prime \prime }$	$F^{\prime \prime }$	$J^{\prime }$	$K_{a}^{\prime }$	$K_{c}^{\prime }$	$F^{\prime }$	ν [MHz]	o.−c.
2	2	0	1	1	1	1	0	9183.705	0.0032
2	2	0	3	1	1	1	2	9185.807	0.0048
2	2	0	2	1	1	1	2	9186.339	0.0028
2	2	0	1	1	1	1	1	9186.714	0.0053
2	2	0	2	1	1	1	1	9187.540	0.0011
2	2	1	2	1	1	0	1	8301.619	−0.0045
2	2	1	2	1	1	0	2	8302.618	−0.0069
2	2	1	3	1	1	0	2	8302.842	0.0016
2	2	1	1	1	1	0	0	8304.456	−0.0068
3	0	3	2	2	1	2	1	8054.940	−0.0003
3	0	3	3	2	1	2	2	8055.124	0.0046
3	0	3	4	2	1	2	3	8055.148	−0.0023
3	2	2	3	2	1	1	2	10765.028	0.0020
3	2	2	4	2	1	1	3	10766.324	0.0094
3	2	2	2	2	1	1	1	10767.030	−0.0006
4	0	4	4	3	1	3	3	10630.422	−0.0002
4	0	4	3	3	1	3	2	10630.509	−0.0009
4	0	4	5	3	1	3	4	10630.584	−0.0004
2	1	1	1	1	0	1	1	7827.541	0.0013
2	1	1	1	1	0	1	0	7828.040	−0.0025
2	1	1	3	1	0	1	2	7828.453	−0.0035
2	1	1	2	1	0	1	1	7829.543	−0.0010
2	1	1	2	1	0	1	2	7829.742	−0.0031
2	2	0	1	1	1	0	1	8592.826	−0.0059
2	2	0	2	1	1	0	1	8593.660	−0.0020
2	2	0	3	1	1	0	2	8594.129	−0.0003
2	2	0	2	1	1	0	2	8594.664	0.0006
2	2	0	1	1	1	0	0	8595.327	−0.0083
2	2	1	2	1	1	1	2	8894.298	0.0003
2	2	1	3	1	1	1	2	8894.510	−0.0033
2	2	1	2	1	1	1	1	8895.506	0.0056
2	2	1	1	1	1	1	1	8895.839	0.0028
3	1	2	2	2	0	2	1	11805.991	−0.0036
3	1	2	4	2	0	2	3	11806.632	−0.0001
3	1	2	3	2	0	2	2	11808.143	0.0021
3	2	1	2	2	1	1	2	11935.818	0.0006
3	2	1	4	2	1	1	3	11937.570	−0.0004
3	2	1	3	2	1	1	2	11937.609	−0.0003
3	2	1	2	2	1	1	1	11937.820	−0.0017
3	2	1	3	2	1	1	3	11938.902	0.0041
3	2	2	2	2	1	2	1	12541.315	0.0015
3	2	2	3	2	1	2	3	12541.912	0.0023
3	2	2	4	2	1	2	3	12541.912	0.0023
3	3	0	3	2	2	0	2	13277.417	0.0034
3	3	0	4	2	2	0	3	13277.810	0.0004
3	3	0	2	2	2	0	1	13278.058	0.0004
4	2	2	3	3	1	2	2	15612.985	−0.0060
4	2	2	5	3	1	2	4	15613.035	0.0072
4	2	2	4	3	1	2	3	15613.524	0.0012
4	2	3	5	3	1	3	4	16398.148	−0.0009
4	2	3	4	3	1	3	3	16399.282	−0.0011
4	3	1	4	3	2	1	3	16340.913	−0.0001
4	3	1	5	3	2	1	4	16341.243	−0.0008
4	3	2	3	3	2	2	2	16911.326	−0.0013
4	3	2	5	3	2	2	4	16911.402	−0.0045
4	3	2	4	3	2	2	3	16911.715	0.0011
